# Effects of functional training on muscle strength, jumping, and functional movement screen in wushu athletes: A systematic review^☆^

**DOI:** 10.1016/j.heliyon.2024.e24087

**Published:** 2024-01-06

**Authors:** Xinzhi Wang, Kim Geok Soh, Nuannuan Deng, Dong Zhang, Shudian Cao, Shamsulariffin Samsudin

**Affiliations:** Department of Sports Studies, Faculty of Educational Studies, University Putra Malaysia, Serdang, 43400, Selangor, Malaysia

**Keywords:** Functional training, Wushu athletes, Muscle strength, Jumping, Functional movement screen

## Abstract

This study aims to analyse the effects of functional training on muscle strength, jumping, and functional movement screen in wushu athletes. Methods: This study followed the guidelines of the Preferred Reporting Items for Systematic Reviews and Meta-Analyses (PRISMA). A systematic search of electronic databases was also conducted, including EBSCOhost, Scopus, PubMed, Web of Science, CNKI, Google Scholar, and Wanfang. The Physiotherapy Evidence Database (PEDro) scale was an effective indicator to evaluate the quality of studies included in the systematic review. Results: This systematic review included 474 participants aged 8–24 years old. The intervention period for most studies was 12 weeks. Among the included studies, 6 focused on muscle strength, 4 on jumping performance, and 11 on functional movement screen. Conclusion: These articles have been analysed, and the positive impact of functional training interventions on muscle strength, jumping, and functional movement screen of wushu athletes has been verified.

**Key Points**

Long-term local strength training may impede the balanced development of small muscle groups, affecting athletes' performance.

Functional training intervention appears to be a feasible strategy to improve specific skills or physical qualities of athletes. Targeted functional training may reduce the probability of sports injuries. Long-term (12–16 weeks) functional training intervention may significantly affect muscle strength, jumping ability, and functional movement screening of wushu athletes.

Further high-quality research is needed to determine the optimal duration, frequency, and intensity of functional training for wushu athletes.

## 1 Introduction

Wushu is a sport that involves all body muscles. The athletic performance level is primarily influenced by the physical fitness of athletes, especially muscle strength [1]. Good physical fitness is crucial for wushu athletes to practice and achieve optimal sports performance [2], especially for the wushu routine [3]. The performance of wushu routine athletes in complex jumping movements depends significantly on their muscle groups’ strength and dynamic balance ability [4]. In wushu-specific training, long-term use of traditional training methods that focus on enhancing local muscle strength can hinder the balanced development of small muscle groups and is not conducive to the improvement of wushu movement quality [[5], [6], [7], [8]]. A single traditional training mode will split the kinematics chain in the training process, thus reducing the efficiency of improving athletes' exceptional sports performance [9,10].

Through scientific and systematic special skill training, athletes' sports performance can be effectively improved [11]. In the early 2000s, functional training began to emerge in the field of sports conditioning [12] that can improve the performance of daily physical activities. This training combines functional and multimodal exercise [13,14]. Compared with the local muscle training of traditional training, functional training pays more attention to the overall training of the human body in sports. Through functional training interventions, a specific skill or physical quality can be improved according to the goal [15]. Unlike traditional training methods, functional training includes aerobic, body-weight, and resistance training [12,13,[16], [17], [18]]. Training intensity varies according to the individual's aim to involve more muscles in activities [19,20]. This approach can be effectively integrated into wushu events. Wushu's various movements reflect stability and explosive power in different sports planes [21]. Dr Arthur Steindler borrowed the kinetic chain theory of Franz Reuleaux, a mechanical engineer, and believed that the human body also has a "closed chain" or "open chain" motion mode [22]. Dr Vladimir Janda proposed the concept of chain reaction, in which the human motion chain system comprises a muscle chain, joint chain, and nerve chain [23]. Completing a good kinetic chain must be based on the complete anatomical structure of the human body and comply with mechanical characteristics [24]. Functional training and a multi-dimensional integrated sports chain system such as nerves, muscles, and bones [25] remarkably improved special sports skills [26].

Functional training has become a recognised global trend in physical health [27]. Functional training is characterised by physical exercise that adapts to joint and muscle coordination and can effectively reduce sports injuries [28,29]. Wushu movements emphasise the coordinated development of muscle strength in all bodily joints, achieving the ultimate difficulty and beauty of the human body through overall physical activity [30]. A complete set of wushu movements requires athletes to maintain high braking force and precise spatial orientation ability [30]. Wushu athletes’ muscle strength is important in achieving excellent sports performance [31]. However, few studies have focused on how to reasonably conduct muscle strength training and evaluate training effects [32]. Meanwhile, jumping ability is also an important factor in sports performance success in competitive sports events [[33], [34], [35], [36], [37]]. Numerous studies have revealed that the performance of vertical jumps directly determines the performance of competitive wushu routines [[38], [39], [40], [41], [42], [43], [44]]. However, these studies usually focus on biomechanical measurements and descriptive studies of jumping movements, and there is relatively little research on wushu training strategies focusing on jumping performance [45].

One study revealed that wushu athletes’ degree and frequency of sports injuries positively correlate with the sports grade [46]. The higher the sports grade, the higher the frequency and degree of sports injuries [46]. If a wushu athlete is restricted in many movements or unable to perform multi-joint movements on multiple planes, the limited motor function will seriously affect their performance [15,47]. Functional movement screen (FMS) is an effective tool to test the quality of athletes' exercise [48]. The body has 7 basic functional movement modes: squat, hurdle, straight lunge, active straight leg lift, shoulder flexibility, torso stability, and rotational stability [49]. FMS scores can find the deficiencies in the subjects' physical flexibility and stability to improve them through targeted functional training and reduce the probability of sports injury [[49], [50], [51], [52], [53]]. Minick and Kate studied the internal reliability of FMS scores and found that 16 items revealed excellent consistency [54]. The study proved that FMS can be widely utilised to screen athletes' sports patterns and assess athletes' potential sports risks [54]. However, few studies have conducted a comprehensive and systematic review and analysis of empirical evidence on the functional movement performance of wushu athletes.

Currently, one systematic review and analysis studied the effects of functional training interventions on cognitive ability [55], muscle strength, balance, agile, and daily living activities in the elderly [56], physical fitness of athletes [57], spring, jumping, functional movement,^58^cardiorespiratory parameters, endurance [58], characterisation of hormonal, metabolic, and inflammatory responses [59], sense of community, satisfaction, and motivation,^61^precedence of injuries [60], and injury risk [50,[61], [62], [63]]. However, no comprehensive study has evaluated the impact of functional training interventions on wushu athletes’ muscle strength, jumping, and functional movement screen. These factors may improve their athletic performance. Therefore, this systematic review explores how functional training affects these factors in wushu athletes.

## 2 Methods

### 2.1 Protocol and registration

The review scheme is designated according to the guidelines of the Preferred Reporting Items for Systematic Reviews and Meta-Analyses (PRISMA) and registered on the website of the International Prospective System Review Register (PROSPERO). The registration number is CRD42023401147.

### 2.2 Search strategy

Before April 2023, famous scholarly databases were utilised to search relevant documents, including EBSCOhost, Scopus, PubMed, Web of Science, CNKI, Wanfang and Google Scholar. The 7 independent databases were strategically searched and queried according to the title, keywords, and abstract. The types of articles include journal articles, master's theses, and doctoral dissertations. The primary keywords considered when collecting relevant research were: ("high-intensity functional training" OR "functional training" OR "functional training exercises" OR "HIFT" OR "functional exercises" OR "cross fit" OR "functional balance training" OR "muscle strength" OR "jumping" OR "jumping performance" OR "functional strength training" OR "functional movement screen" OR "functional movement pattern" OR "functional movement skills" OR "FMS") AND ("wushu" OR "martial arts" OR "wushu routine" OR "traditional wushu routine" OR "competitive wushu routine" OR "wushu exercise" OR "wushu sports" OR "wushu movement" OR "wushu athlete"). Manual search and review of relevant article reference lists to supplement.

### 2.3 Eligibility criteria

This study employed the PICOS model for the article's screening criteria [64]. PICOS includes (1) population; (2) intervention; (3) comparison; (4) results; and (5) study design. The publications summarised in the study must fulfil the following inclusion (Table 1) and exclusion criteria:

**Table 1:** PICOS eligibility inclusion criteria.

Category	Inclusion Criteria
Population	Healthy wushu athletes (male/female)
Intervention	Functional training
Comparative intervention	Two or multiple groups of controlled trials
Outcome	At least 1 measure related to muscle strength or jumping or functional movement screen
Study design	Randomised controlled trails

This screening adhered to the date of database creation. This systematic review included the following exclusion criteria: (1) Studies with no emphasis on functional training (for example, strength training or agility training only); (2) Books, chapters, other horizontal studies, and publications unrelated to functional training; (3) Studies including no future research, retrospective research, abstracts, case reports, reviews, or patents; and (4) No-randomized controlled trials.

### 2.4 Study selection

**Fig. 1:**
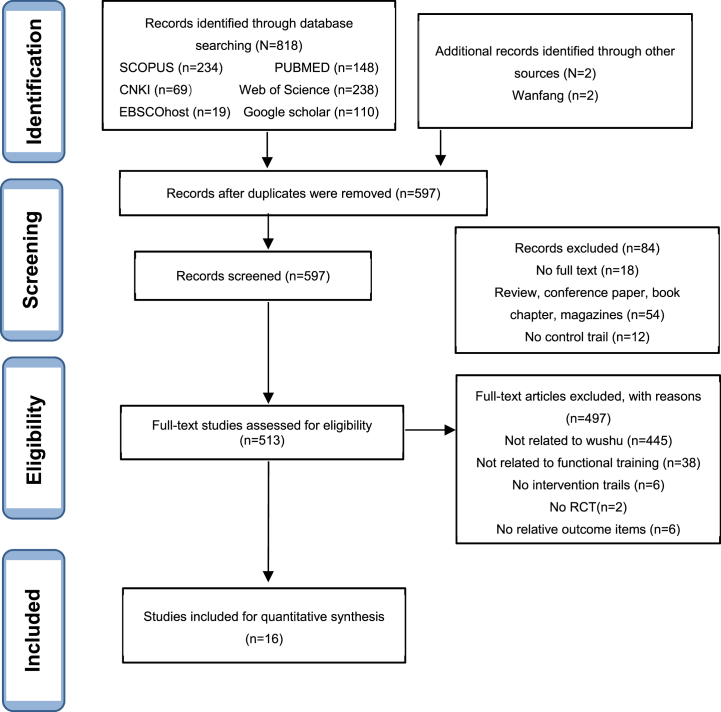
Flow chart of PRISMA article screening.

First, 2 independent reviewers screened and retrieved all the research related to the subject according to the inclusion and exclusion criteria and deleted the duplicates and irrelevant articles. Then, according to the PICOS model, they reviewed the full text of the potentially qualified papers. If there were any reasonable differences during the screening process, continue to discuss with the third reviewer until a consensus was reached. Finally, the system review included 16 articles that met the inclusion requirements (Fig. 1).

### 2.5 Data items and data extraction

This study screened all relevant articles, identified the appropriate articles based on the inclusion and exclusion requirements, and studied the measurement indicators such as the squat, bench press, jump, and functional movement screen scores. The following details were extracted: (1) Author and year of publication; (2) Research design; (3) Demographic characteristics of the sample (sample size, gender, age, height, and weight); (4) Intervention characteristics (type, frequency, and length of intervention); (5) Measurement indicators; and (6) Test outcomes.

### 2.6 Study quality assessment

The Physiotherapy Evidence Database (PEDro) scale was an effective indicator to evaluate the quality of the studies included in the systematic review. The PEDro scale primarily comprises 4 aspects: random process, blind technology, group comparison, and data analysis. Independent raters provide points for answering "Yes" (1) or "No" (0). The quality of the associated methodology is higher the higher the score. The PEDro scale's maximum score was achieved by 10 out of the eleven items.

## 3 Results

This review includes 16 studies on the effects of functional training on athletes' muscle strength, jumping performance, and functional movement screen. These studies were published between 2017 and 2022. Table 3 summarises these studies' main characteristics. Fig. 1 describes the process of research screening.

**Table 2:** Methodological quality assessment for inclusion studies.

Study	Eligibility Criteria	Random Allocation	Allocation Concealment	Baseline Comparability	Blind Participants	Blind Therapist	Blind Assessor	Follow-Up	Intention to Treat Analysis	Between Group Comparisons	Point Measure and Variability	Total PEDro Score
Cai Z. (2020)	1	1	0	1	0	0	0	1	0	1	1	6
Chen C. (2020)	1	1	0	1	0	0	0	1	1	1	1	7
Fan W. (2020)	0	0	0	1	0	0	0	1	1	1	1	5
Fang C. (2018)	0	0	0	1	0	0	0	1	0	1	1	4
Liu H. (2018)	1	1	0	1	0	0	0	1	0	1	1	6
Liu X. (2018)	1	1	0	1	0	0	0	1	0	1	1	6
Song Y. (2015)	1	1	0	1	0	0	0	1	0	1	1	6
Sun T. (2018)	1	1	0	1	0	0	0	1	0	1	1	6
Sun Y. (2021)	0	0	0	1	0	0	0	1	0	1	1	4
Wang J. (2019)	1	1	0	1	0	0	0	1	0	1	1	6
You K. (2020)	1	0	0	1	0	0	0	1	0	1	1	5
Zhang C. (2022)	1	1	0	1	0	0	0	1	0	1	1	6
Zhang D. (2021)	0	1	0	1	0	0	0	1	0	1	1	5
Zhang J. (2021)	1	1	0	1	0	0	0	1	0	1	1	6
Zhou K. (2018)	1	0	0	1	0	0	0	1	0	1	1	5
Zhu H. (2017)	1	1	0	1	0	0	0	1	0	1	1	6

**Table 3:** Summary of the studies’ characteristics included in this review.

Study	Design	Participants characteristics	Frequency/Session duration (Mins)/Training duration	Comparator	Outcomes
Cai Z. (2020)	pre-post test	EG (n = 10), CG (n = 10) Gender: 13 M, 7F Age: 16–19 years; height: 153–175 cm; body mass: 46–78 kg Professional athletes	Freq.: 5 times/week, time: 30min, length: 8weeks	EG: Daily Wushu Routine Training and Functional Movement Correction Training Program CG: Daily Wushu Routine Training	FMS↑
Chen C. (2020)	pre-post test	EG (n = 30), CG (n = 30) Gender: 30 M, 30F Age: 1 EG: 43 ± 0.78 years; height: 140.28 ± 3.72 cm; body mass: 33.31 ± 2.27 kg Junior athletes	Freq.: 3 times/week, time: 60 min, length: 12weeks	EG: Functional Agility Training Program CG: Traditional Agility Training Program	Jump scores (HJT)↑
Fan W. (2020)	pre-post test	EG (n = 20), CG (n = 20) Gender: 20 M, 20F Age: 15.8±EG: 2 years; height: 174.6 ± 9.63 cm; body mass: 63.3 ± 10.3 kg College athletes	Freq.: 3 times/week, time: 45min, length: 8weeks	EG: Daily 24-style Taijiquan Training and Functional Training Program CG: Daily 24-style Taijiquan Training	FMS↑, Jump scores (SLJ)↑
Fang C. (2018)	pre-post test	EG (n = 10), CG (n = 10) Gender: 20F Age: 19.50 ± 2.27 years; height: 168.00 ± 5.58 cm; body mass: 62.30 ± 8.41 kg; BMI: 20.15–23.93 kg/m^2^ Elite athletes	Freq.: 3 times/week, time: 30min, length: 8weeks	EG: Functional Movement Correction Training Program CG: Regular Training Program	FMS↑
Liu H. (2018)	pre-post test	EG (n = 6), CG (n = 6) Gender: 12 M Age: 19.00±EG: 41 years; height: 174.57 ± 5.13 cm; body mass: 74.92±1 EG: 94 kg Professional athletes	Freq.: 2 times/week, time: 20min, length: 12weeks	EG: Functional Training Program CG: Traditional Training Program	Muscle Strength (1MSU, 1MSKAR, TSB)↑
M, male; F, female; NR, no record; EG, experiment group; CG, control group; Freq, frequency; FMS, functional movement screening; HJT, hexagonal jump test; SLJ, standing long jump; 1 MSU, 1 min sit-ups; 1MSKAR, 1min simultaneous knee and abdominal raise; TSB, throwing solid ball practice; VJTH, vertical jump touch high; STJ, standing triple jump; RUOLHJ, Run-up one-leg high jump; FPJTH, former place jump to touch height; OLSF360°, Outward Leg Swing in Flight at 360°; WWK360°, Whirl Wind Kick at 360°; FF, Flying feet; SS, Side somersault; JDAC, jumping difficulty action cohesion; 1RM, one-repetition maximum; PU, push up; BP, bench press; BLT, Badminton Long Throw; EMG, electromyography.					
Study	Design	Participants characteristics	Frequency/Session duration (Mins)/Training duration	Comparator	Outcomes
Liu X. (2018)	pre-post test	EG (n = 20), CG (n = 20) Gender: 40 M Age: 2 EG: 30 ± 0.733 years; height: 177.85 ± 3.950 cm; body mass: 7 EG: 325 ± 5.768 kg College athletes	Freq.: 2 times/week, time: 20min, length: 12weeks	EG: Body Function Movement Preparation Training Program CG: Traditional Preparation Activity Program	Muscle Strength (1 MSU) ↑, FMS↑
Song Y. (2015)	pre-post test	EG (n = 9), CG (n = 9) Gender: 10 M, 8F Age: 2 EG: 5±EG: 14 years; height: 172±EG: 87 cm; body mass: 72.4 ± 19.81 kg Professional athletes	Freq.: 3 times/week, time: 55min, length: 12weeks	EG: Functional Training Program CG: Traditional Training Program	Jump scores (VJTH, SLJ, STJ, JDAC) ↑
Sun T. (2018)	pre-post test	EG (n = 12), CG (n = 12) Gender: 24 M Age: 9.50±EG: 31 years; height: 139.75 ± 5.39 cm; body mass: 3 EG: 25 ± 4.40 kg Junior athletes	Freq.: 3 times/week, time: 45 min, length: 12weeks	EG: Functional Training Program CG: Traditional Training Program	Muscle Strength (RUOLHJ, FPJTH) ↑, FMS↑Jump scores (FF, OLSF360°, WWK360°, SS) ↑
Sun Y. (2021)	pre-post test	EG (n = 4), CG (n = 4) Gender: 4 M, 4F Age: 17. 9±EG: 40 years; height: 163. 86 ± 5.2 cm; body mass: 59.00 ± 2.16 kg; BMI: 17.2-2 EG: 4 kg/m^2^ Professional athletes	Freq.: 3 times/week, time: 60min, length: 10weeks	EG: Functional Movement Correction Training Program CG: Traditional Physical Training Program	FMS↑, Muscle Strength (1RM Squat↑ BP↔)
Wang J. (2019)	pre-post test	EG (n = 20), CG (n = 20) Gender: 24 M, 16F Age: 13–24 years; height: 157–178 cm; body mass: 49–74 kg; BMI: 19.6–26.0 kg/m^2^ Professional athletes	Freq.: 2 times/week, length: 12weeks	EG: Daily Wushu technical and tactical training and functional movement correction training program CG: Daily Wushu Technical and Tactical Training	FMS↑
M, male; F, female; NR, no record; EG, experiment group; CG, control group; Freq, frequency; FMS, functional movement screening; HJT, hexagonal jump test; SLJ, standing long jump; 1 MSU, 1 min sit-ups; 1MSKAR, 1min simultaneous knee and abdominal raise; TSB, throwing solid ball practice; VJTH, vertical jump touch high; STJ, standing triple jump; RUOLHJ, Run-up one-leg high jump; FPJTH, former place jump to touch height; OLSF360°, Outward Leg Swing in Flight at 360°; WWK360°, Whirl Wind Kick at 360°; FF, Flying feet; SS, Side somersault; JDAC, jumping difficulty action cohesion; 1RM, one-repetition maximum; PU, push up; BP, bench press; BLT, Badminton Long Throw; EMG, electromyography.					
Study	Design	Participants characteristics	Frequency/Session duration (Mins)/Training duration	Comparator	Outcomes
You K. (2020)	pre-post test	EG (n = 24), CG (n = 10) Gender: 21 M, 13F Age: 19.7 ± 4.4 years; height: 17 EG: 2 ± 4.4 cm; body mass: 63.1 ± 7.0 kg Professional athletes	Freq.: 3 times/week, time: 60min, length: 12weeks	EG: Daily Wushu Routine Training and Functional Movement Correction Training Program CG: Daily Wushu Routine Training	FMS↑
Zhang C. (2022)	pre-post test	EG (n = 10), CG (n = 10) Gender: 10 M, 10F College athletes	Freq.: 2 times/week, time: 45 min, length: 12weeks	EG: Functional Training Program CG: Traditional Training Program	FMS↑
Zhang D. (2021)	pre-post test	EG (n = 12), CG (n = 12) Gender: 24 M Age: 24.42 years; height: 169.42 cm; body mass: 6 EG: 83 kg Professional athlete	Freq.: 3 times/week, time: 60min, length: 16weeks	EG: Functional Training Program CG: Traditional Strength Training Program	Muscle Strength (Power Clean↑, Burpees↑, BLT↔, PU↔)
Zhang J. (2021)	pre-post test	EG (n = 7), CG (n = 7) Gender: 14 M Age: 20.57 ± 0.78 years; height: 175.91 ± 4.86 cm; body mass: 69.51 ± 7.02 kg Professional athletes	Freq.: 3 times/week, time: 45min, length: 12weeks	EG: Functional Training Program CG: Traditional Agility Training Program	Muscle strength (EMG tester) ↑
Zhou K. (2018)	pre-post test	EG (n = 20), CG (n = 20) Gender: NR Age: 2 EG: 90 ± 0.86 years; height: 165.4 ± 2.22 cm; body mass: 65.20 ± 3.08 kg Professional athletes	EG: Freq.: 5 times/week, time: 90min, length: 4weeks 2.Freq.: 3 times/week, time: 90min, length: 6weeks	EG: Functional Movement Correction Training Program and Injury prevention training CG: Injury prevention training Program	FMS↑
Zhu H. (2017)	pre-post test	EG (n = 30), CG (n = 30) Gender: 40 M, 20F Age: 19.40 ± 0.46 years; height: 170.70 ± 15.39 cm; body mass: 60.7 ± 16.60 kg College athletes	Freq.: 1 times/week, time: 90min, length: 16weeks	EG: Functional Training Program and Wushu Special Quality Training CG: Wushu Special Quality Training	FMS↑
M, male; F, female; NR, no record; EG, experiment group; CG, control group; Freq, frequency; FMS, functional movement screening; HJT, hexagonal jump test; SLJ, standing long jump; 1 MSU, 1 min sit-ups; 1MSKAR, 1min simultaneous knee and abdominal raise; TSB, throwing solid ball practice; VJTH, vertical jump touch high; STJ, standing triple jump; RUOLHJ, Run-up one-leg high jump; FPJTH, former place jump to touch height; OLSF360°, Outward Leg Swing in Flight at 360°; WWK360°, Whirl Wind Kick at 360°; FF, Flying feet; SS, Side somersault; JDAC, jumping difficulty action cohesion; 1RM, one-repetition maximum; PU, push up; BP, bench press; BLT, Badminton Long Throw; EMG, electromyography.					

### 3.1 Study selection

First, a search of different electronic databases presented 820 potential articles (SCOPUS = 234, PubMed = 148, CNKI = 69, Web of Science = 238, EBSCOhost (Sport Discus) = 19, Google scholar = 110, Wanfang = 2). After excluding duplicates (223), the titles and abstracts of 597 articles were screened, and 513 articles were identified as potentially eligible, followed by a secondary screening. Sixteen articles were eligible for inclusion and considered for inclusion data synthesis for article systems.

### 3.2 Study quality assessment

Table 2 presents information about the quality score of the PEDro scale. The average score of the included studies on the PEDro scale is 5.56 (range 4–7). This scale suggests that no studies met all the quality standards of the PEDro scale. All studies were deducted points due to the criteria related to allocation concept, blind participants, blind therapists, and blind assessors. However, all studies met the baseline compatibility, follow-up, point measure, and variability criteria. Only 2 studies revealed that all subjects received treatment [5,65]. Ensuring all subjects receive treatment can avoid grouping imbalance between treatment and control groups.

### 3.3 Participant characteristics

Table 3 presents the participant characteristics in 16 studies that met the inclusion criteria and the information on functional training interventions, primarily including the following parts. (1) Gender classification: Five of the 16 articles studied male athletes [7,[66], [67], [68], [69]]. One studied female athletes [70]. Nine mixed studies focused on male and female athletes [5,6,8,21,65,[71], [72], [73], [74]], but 1 study did not report information on gender [75]. (2) Sample size: In 16 studies, the total number of subjects was 474, ranging from 8^74^ to 60,^5,76^ with a median of 24 [7]. The average sample number was 29.18. (3) Age: General studies indicated the participants' age, except one [73]. The age range is 8^7^ to 24^70^, with an average age of 16. (4) Height, weight, and body mass index (BMI): Most studies explained the subjects' height and weight information [7,66,67,70], except one [73]. Three studies reported the subjects' BMI [21,70,72]. (5) Wushu training background: Ten of the 16 studies described the tested athletes' training years [5,6,69,71], while the other 6 studies did not [65,67,[72], [73], [74], [75]]. For the convenience of statistical analysis, the wushu athletes’ training duration is calculated in months, and the range is 24–84 months.

### 3.4 Intervention characteristics

The 16 included studies were based on the intervention characteristics of the following 3 aspects: length of intervention, duration of a single intervention, and frequency of intervention. (1) Intervention duration: The most extended intervention period was 16 weeks (2 studies) [68,74]. Three studies presented a study period of 8 weeks [65,71,74]. Two studies presented a study period of 10 weeks [72,75], and nine studies presented a study period of 12 weeks [[5], [6], [7], [8],^21^,^66^,^67^,^73^,^73^]. (2) Duration of a single intervention: Most studies reported the duration of a single intervention [7,65,69,70], but one did not [21]. The analysis of the 16 included studies discovered that the duration of a single intervention ranged from 20 min [66,66] to 90 min [75]. (3) Intervention frequency: All included studies reported the frequency of intervention [8,70,73]. The lowest frequency was once a week [74], and most studies intervened 3 times a week [7,65,68,69].

### 3.5 Outcome

#### 3.5.1 Effect of functional training on muscle strength of wushu athletes

Six of the 16 studies evaluated the effect of functional training on wushu athletes' muscle strength. The evaluation methods and test tools utilised in these studies include 1 min sit-up [66,67], 1 min simultaneous knee and arbitrary raise, throwing solid ball test [66], run-up one-leg high jump, form place jump to touch height [7], horizontal push, and push-up. The studies also employed Swiss ball square, badminton throw, power clean, square, bench press, single-leg four-step jump, vertical and horizontal support [68], and electromyography test [69]. The subjects were elite wushu athletes [68,70], professional wushu athletes [6,8,69,71,72,75], college players [65,67,73,74], and junior players [5,7]. Four studies reported the positive effect of functional training interventions on wushu athletes’ muscle strength [7,66,67,69]. Two studies reported that the number of power clean [68], burpees [68], and squats [72] dramatically enhanced after the intervention of functional training. However, there was no significant difference between pull-up [68], badminton long-throw [68], and bench press [72] records before and after the experimental intervention.

#### 3.5.2 Effect of functional training on jumping performance of wushu athletes

Four of the 16 studies report on the effect of functional training interventions on jumping performance [[5], [6], [7],^65^]. One study reported the performance of wushu athletes' jump difficulty movements (such as side somersaults and whirlwind feet) [7]. One reported the fast jump performance [5], and one only reported the standing long jump performance [65]. One study reported the vertical jump, standing long jump, and the cohesion ability to jump difficult movements [6].

After the functional training (FT) intervention, comparing the pre-test and post-test results reveals that the performance of the FT group's hexagonal jump test improved significantly [5]. After the 12-week intervention, the test reveals a significant improve in the experimental group athletes' success rate in completing the difficult jump movement [7]. Similarly, another study found that the 12-week intervention experiment significantly impacted the connection quality of the difficult jump movement of wushu athletes [6].

#### 3.5.3 Effect of functional training on functional movements of wushu athletes

Eleven of the 16 studies reported on the effect of functional training interventions on athletes' functional movement screen scores. Before the experiment, there was no difference in FMS scores between FTG and TTG, FTG, and CG. After the functional training intervention experiment, the test found that the FMS scores of FTG were significantly improved compared with CG or TTG [7,67,70,71]. According to the FMS-related research included in this system review, the change in FMS total score is primarily affected by the scores of hurdle step, straight lunge, torso stability, and rotational stability [7,67]. According to the evaluation results of FMS, after the functional training intervention, there was no significant difference between the experimental group in the squat [7], shoulder flexibility [67], and active straight leg lift [8] before and after the test. The studies reported that after the functional training intervention, the average score of all FMS test indicators in the experimental group increased by 7 points, indicating that the functional training intervention significantly improved the essential sports ability of wushu athletes [72].

## 4 Discussions

This systematic review seeks to comprehensively summarise the effect of functional training on muscle strength, jumping, and functional movement screen of wushu athletes. This study differs from previous research on the effects of functional training on athletes. Specifically, it focuses on wushu athletes and evaluates the effects of functional training on muscle strength, jumping performance, and functional movement. The key conclusions of this systematic review suggest that functional training may be an effective method for improving wushu athletic performance. However, the research on functional training interventions summarised in this systematic review has significant differences in subject characteristics (age, sex, height, and weight) and physical health components. The included research also has significant differences in the weekly training frequency, total duration of functional training interventions, single training, and measurement results. According to the study's significant results, functional training may positively impact wushu athletes' muscle strength, jumping, and functional movement screen.

### 4.1 Effect of functional training on muscle strength of wushu athletes

Numerous factors influence jumping ability, including the ratio of fast-twitch and slow-twitch muscle fibres [76]. However, jumping height depends mainly on the strength of the muscles in the lower limbs [77]. Functional training significantly improved male wushu athletes’ lower limb muscle strength (run-up one-leg high jump, Swiss ball square), waist and abdominal core muscle strength (1 min sit-ups,1 min simultaneous knee and abdominal muscle, power clean), and upper limb muscle strength (throwing a solid ball) [7,[66], [67], [68]]. Several articles have investigated the impact of functional training on muscle strength among mixed-gender wushu athletes [72]. To date, no research has examined the impact of functional training interventions on the muscle strength of female wushu athletes. Further research is needed to investigate this.

Additionally, the results indicate that the effectiveness of functional training in improving muscle strength may vary depending on the training program's type, frequency, and duration. High-intensity and high-volume functional training interventions significantly improved muscle strength among wushu athletes [69], while low-intensity and low-volume programs may also be effective [66,67,71]. Compared with the training group, young wushu routine athletes' lower limb muscle strength was tested after 12 weeks of functional training interventions. In this study, the muscle strength of lower limbs in the FT group and the traditional training (TT) group improved after 12 weeks, but the muscle strength parameters in the FT group increased significantly [7]. One study revealed that FT and TT groups were trained in different ways simultaneously. After the 16-week training, FT athletes' primary, general, and unique sports abilities significantly improved, especially in core stability and muscle strength [68]. Additionally, the study of 12 weeks of functional training 2 days a week and once for 20 min revealed no significant difference between the experimental data of the fourth week and before the experiment. The experimental data of the eighth week had a statistical difference compared with the fourth week. There was also a significant improve between the experimental data after 12 weeks of training and the eighth week [66]. Therefore, long-term functional training interventions are expected to significantly improve wushu athletes' muscle strength.

### 4.2 Effect of functional training on jumping performance of wushu athletes

Jumping is a physical ability that athletes must have [78]. Two studies indicated the positive effect of functional training interventions experiments on the great difficulty jump in the wushu project [6,7]. After 12 weeks of functional training interventions, the cohesion ability of the high-difficulty jumping significantly improved, though the effect of functional training on female athletes is different. There was no statistical difference between the performance of the female FT and control groups in the standing long jump [6]. Another study found that after the functional training intervention, the performance of the FT and control group in the vertical jump movement improved, but the FT group improved significantly. The average score of the FT group athletes in the prosperous times of flying feet increased by 1.67, with an increased rate of 33.4 %. The average result of difficulty movements of rotation increased by 0.78, with an increased rate of 15.8 %. The average result of side somersault increased by 3.58, with an increased rate of 71.6 % [7]. Therefore, the experimental group receiving functional training interventions may improve performance more than the control group.

A study reported the effect of functional training interventions on the coordination ability of wushu athletes [5]. Under the same training environment, training time, and training load, male and female athletes' hexagonal jump test data significantly improved. The research proved that the hexagonal jump is very suitable for testing the coordination ability of wushu athletes. When jumping at high speed and in multiple directions, the athletes must maintain high sensitivity and react quickly in case of physical instability or sudden conditions, which can well reflect the physical quality and movement rhythm of wushu athletes [5]. Therefore, the analysis of the selected studies indicates that functional training might be a good way to enhancing wushu jumping ability. Jumping ability is crucial in wushu competition, including jump height, jump speed, and aerial technique. This systematic review is consistent with previous research that reports functional training can improve the jumping ability of athletes from different sports [[79], [80], [81], [82]].

### 4.3 Effect of functional training on functional movements screen of wushu athletes

Functional movement screen is a tool for assessing basic individual exercise patterns [83,84], screening athletes for possible injuries and muscular weaknesses during exercise, and providing personalised and functional recommendations for improving athletes' sports performance [28]. Based on previous research, athletes with low FMS scores are at a higher risk of sustaining injuries [85,86], especially those involved in high-intensity sports such as wushu [87]. Research has analysed the FMS scores of 20 high-level wushu athletes and found that squatting has the lowest FMS score. When squatting, athletes are assisted by compensatory actions such as reverse back flexion, excessive forward-leaning, arched back, and asymmetric centre of gravity transfer due to insufficient neuromuscular control ability [71]. Functional training effectively reduces the risk of sports injuries and improves the FMS scores of different populations [88,89]. After 8 weeks of functional training, the FMS score of the experimental group was significantly higher than that of the control group, and the physical function significantly improved. The subjects were more satisfied with the functional training intervention methods than with traditional training methods [65].

The study analysed the impact of the 12-week functional training intervention experiment on the FMS scores of wushu athletes by time stage [21]. The FMS scores of subjects after the 4 weeks functional training intervention were not statistically significant. After 8 weeks, the FMS scores of the experimental intervention group were significantly higher than the control group. After 12 weeks, the control group's FMS scores displayed a downward trend, while the intervention group's scores were significantly higher than before [21]. Seven studies tested FMS scores to determine athletes' basic movement abilities and targeted functional correction training programs [7,8,67,70,[72], [73], [74]]. The research found that adding functional corrective training to daily training programs can reduce the incidence of sports injuries [74,75] and improve participants' training satisfaction [67]. Wushu coaches and practitioners will find considerable value in the findings of this systematic review. They can employ focused exercises to lower their risk of injury and enhance the quality of their movements.

## 5 Limitations

This systematic review adhered to the PRISMA reporting guidelines and utilised the PEDro scale to appraise the quality of each study included. It provided relevant evidence on the effects of functional training interventions on muscle strength, jump performance, and functional movements in wushu athletes while ensuring fair quality assessment. However, the measurement tools used to evaluate jump performance varied significantly among these studies, which hindered comparability between research results. Therefore, future studies should use standardised measurement tools to ensure comparability and reliability of research results. Additionally, manual search and review of relevant article reference lists to supplement. Some articles indirectly related to martial arts athletes may have been missed since they were not explicitly mentioned in the keywords. Furthermore, the number of studies incorporated in this systematic review was restricted, and the sample sizes were generally small, which may have limited the statistical power of the research findings. Moreover, this systematic review may have overlooked some relevant studies. For instance, wushu research based on computer algorithms and capture technology may evaluate FMS in athletes more scientifically, but these studies were not included.

## 6 Conclusions

This systematic review reports that functional training interventions may positively impact the muscle strength, jumping performance, and functional movement screen of Wushu athletes. The findings of this study will help demonstrate the importance of functional training for Wushu athletes. Researchers will be able to use this study to plan different training approach to find the optimal way of physical fitness training. The studies included in this systematic review varied in their training content, intervention frequency, duration, and single intervention session duration, which may have influenced the results. Future research should focus on identifying the specific components of functional training that are most effective in improving the muscle strength, jumping performance, and functional movement screen of Wushu athletes and whether improvements result in better athletic performance and lower injury risks.

## Data availability statement

Data included in article/supp. Material/referenced in article.

## Funding

This research received no external funding.

## Ethical approvals

Not required.

## Additional information

No additional information is available for this paper.

## CRediT authorship contribution statement

**Xinzhi Wang:** Writing - review & editing, Writing - original draft. **Kim Geok Soh:** Supervision. **Nuannuan Deng:** Validation. **Dong Zhang:** Software. **Shudian Cao:** Supervision. **Shamsulariffin Samsudin:** Supervision.

## Declaration of competing interest

The authors declare that they have no known competing financial interests or personal relationships that could have appeared to influence the work reported in this paper.
